# A rare case of hepatic epithelioid hemangioendothelioma diagnosed by EUS-guided fine-needle biopsy (with videos)

**DOI:** 10.1097/eus.0000000000000111

**Published:** 2025-04-23

**Authors:** Yating Wang, Beiyao Zhang, Dongqiang Zhao

**Affiliations:** Department of Gastroenterology, The Second Hospital of Hebei Medical University, Hebei Key Laboratory of Gastroenterology, Hebei Institute of Gastroenterology, Hebei Clinical Research Center for Digestive Diseases, Shijiazhuang, Hebei Province, China.

A 35-year-old man was admitted to our department, with a 9-day history of jaundice. Preadmission liver function tests indicated hepatocellular damage and cholestasis, and abdominal ultrasound revealed multiple heterogeneous echoic masses in the liver. Tumor marker tests showed the following: alpha fetoprotein (AFP), 37.05 ng/mL; carcinoembryonic antigen (CEA), 4.47 ng/mL; carbonic anhydrase (CA)-125, 46.14 U/mL; and CA-199, 385.37 U/mL. Abdominal contrast-enhanced CT suggested multiple hypodense lesions in the liver [Figure 1]. Abdominal magnetic resonance imaging (MRI) revealed multiple intrahepatic lesions with abnormal signals [Figure 2A–B], some subcapsular, with hepatic capsular retraction. Diffusion-weighted imaging (DWI) showed restricted diffusion with pronounced hyperintensity [Figure 2C]. On contrast-enhanced imaging, some lesions showed peripheral ring enhancement in the arterial phase, followed by centripetal enhancement during the venous and delayed phases, with prominent enhancement in the late delayed phase [Figure 2D–G]. These findings are highly suggestive of hepatic epithelioid hemangioendothelioma (HEHE). Given the location of the lesions, EUS-guided fine-needle biopsy (EUS-FNB) was performed for definitive diagnosis. EUS (GF-UCT260, Olympus) revealed multiple quasi-circular hypoechoic lesions in the left lobe of the liver, most at the liver margin, measuring approximately 15 mm × 12 mm, with well-defined borders and heterogeneous internal echoes [Figure 3A, Video 1]. Color Doppler flow imaging showed no significant blood flow signals within the lesions [Figure 3B]. Elastography displayed blue-green coloration [Figure 3C], and contrast-enhanced EUS imaging revealed weak enhancement [Figure 3D]. A 22-gauge FNB needle (EUS-22-1-N, Micro-Tech) was used to access the liver lesions via the stomach, with 2 passes made [Figure 4, Video 2]. No complications occurred post-biopsy. Immunohistochemistry showed positivity for CD31, CD34, cytokeratin pan (CKpan), erythroblastosis transformation-specific regulated gene (ERG), and friend leukemia virus integration 1 (FLi-1), with a Ki-67 index of 5% [Figure 5]. The final pathological diagnosis was HEHE. Given the unresectable hepatic lesions without distant metastases, liver transplantation was recommended.

**Supplementary Videos**

Video 1: EUS imaging showed the location and characteristics of the hepatic lesions in the left lobe of the liver; Video 2: EUS-FNB procedure with a 22-gauge FNB needle. Videos are only available at the official website of the journal (www.eusjournal.com).

HEHE is a rare neoplasm of vascular origin with a variable course.^[1]^ Its progression ranges from long-term survival without treatment to rapid deterioration and fatal outcomes. Clinically, it is often an incidental finding, with symptoms ranging from nonspecific signs to advanced liver failure.^[2]^ The definitive diagnosis of HEHE relies on histopathology, and this case underscores the critical role of EUS-FNB as a crucial tool for obtaining definitive tissue samples.

**Figure 1:**
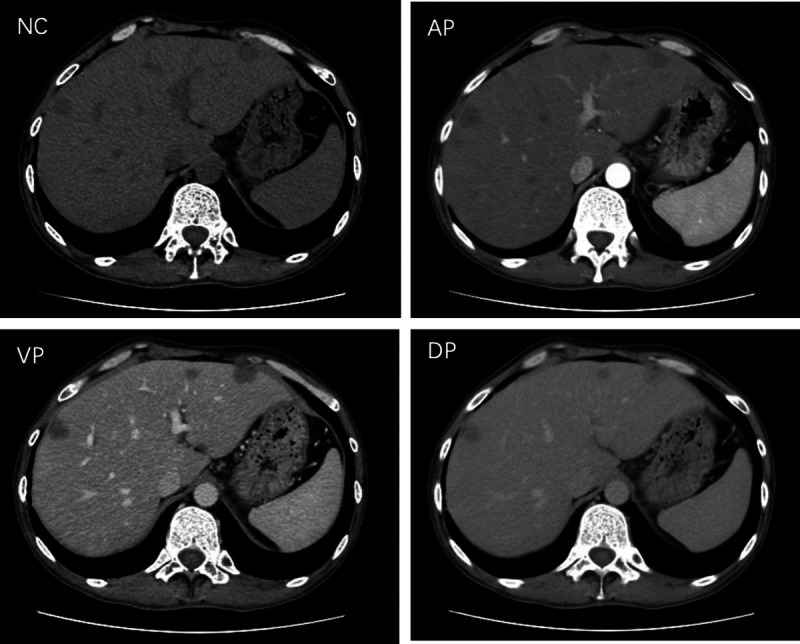
Abdominal contrast-enhanced CT suggested multiple hypodense lesions in the liver, with mild enhancement on the contrast phase. Small blood vessels appeared to pass through the lesions during the arterial phase. NC: Noncontrast; AP: Arterial phase; VP: Venous phase; DP: Delayed phase.

**Figure 2:**
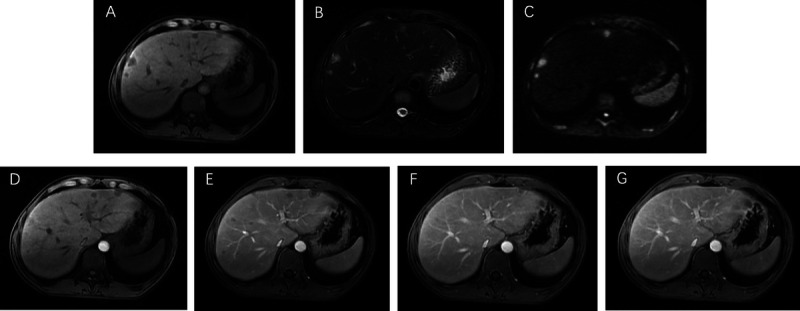
Abdominal MRI revealed multiple intrahepatic lesions: hypointense on T1WI (A) and hyperintense on T2WI (B). DWI (C) showed restricted diffusion with marked hyperintensity. Contrast-enhanced imaging revealed peripheral ring enhancement in the arterial phase (D), centripetal enhancement during the portal venous (E) and delayed phases (F), and prominent enhancement in the late delayed phase (G).

**Figure 3:**
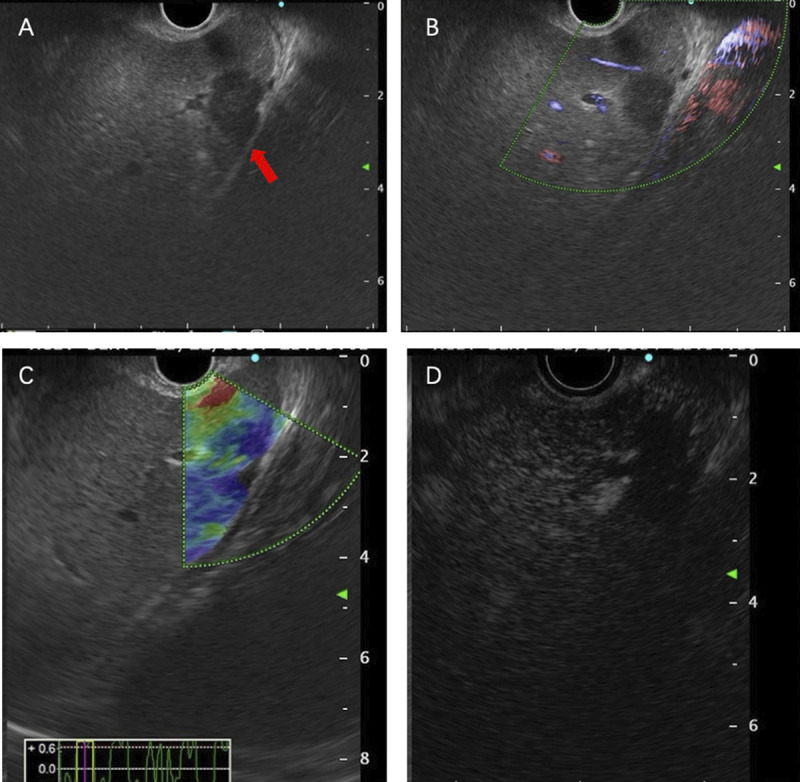
EUS (A) showed a quasi-circular hypoechoic lesion in the left lobe of the liver, approximately 15 mm × 12 mm. Color Doppler flow imaging (B) showed no significant blood flow signals. Elastography (C) displayed blue-green coloration. Contrast-enhanced EUS imaging (D) revealed weak enhancement.

**Figure 4:**
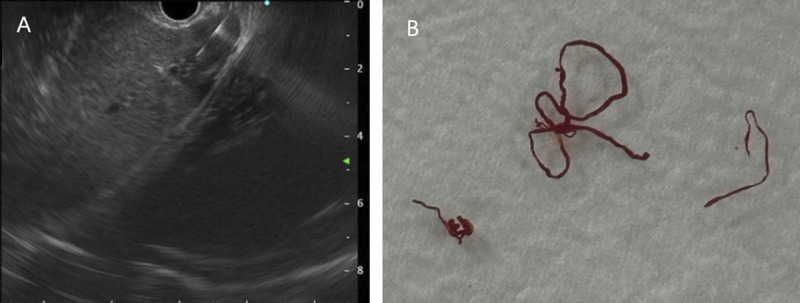
EUS-FNB (A) was performed via the stomach with 22-gauge FNB needle. Tissue samples (B) were shown.

**Figure 5:**
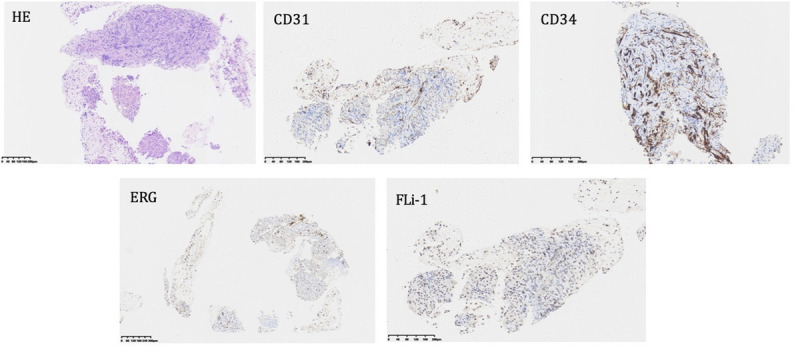
Hematoxylin and eosin staining (HE), and immunostaining of CD31, CD34, ERG, and Fli-1 of the sample were shown. ERG, erythroblastosis transformation-specific regulated gene (ERG); FLi, friend leukemia virus integration 1.

## Acknowledgments

None.

## Source of Funding

None.

## Ethical Approval

None declared.

## Informed Consent

We obtained written informed consent from the patient to publish this article.

## Conflict of Interest

The authors declare no conflict of interest for this article.

## Author Contributions

Yating Wang reviewed the literature and contributed to manuscript writing and video producing, Beiyao Zhang cared for the patient and prepared the figure. Dongqiang Zhao was the operator who performed the EUS-FNB and was responsible for the conceptualization and supervision. All authors issued final approval for the version to be submitted.
